# Reliability of an Inertial Measurement System Applied to the Technical Assessment of Forehand and Serve in Amateur Tennis Players

**DOI:** 10.3390/bioengineering12010030

**Published:** 2025-01-02

**Authors:** Lucio Caprioli, Cristian Romagnoli, Francesca Campoli, Saeid Edriss, Elvira Padua, Vincenzo Bonaiuto, Giuseppe Annino

**Affiliations:** 1 Sports Engineering Laboratory, Department of Industrial Engineering, University of Rome Tor Vergata, 00133 Rome, Italy; francesca.campoli@uniroma5.it (F.C.); saeid.edriss@alumni.uniroma2.eu (S.E.); vincenzo.bonaiuto@uniroma2.it (V.B.); 2 Department of Human Science & Promotion of Quality of Life, San Raffaele Rome University, 00166 Rome, Italy; 3 Human Performance Laboratory, Centre of Space Bio-Medicine, Department of Medicine Systems, University of Rome Tor Vergata, 00133 Rome, Italy; giuseppe.annino@uniroma2.it

**Keywords:** tennis technique, tennis performance, kinematics, biomechanics, IMU

## Abstract

Traditional methods for evaluating tennis technique, such as visual observation and video analysis, are often subjective and time consuming. On the other hand, a quick and accurate assessment can provide immediate feedback to players and contribute to technical development, particularly in less experienced athletes. This study aims to validate the use of a single inertial measurement system to assess some relevant technical parameters of amateur players. Among other things, we attempt to search for significant correlations between the flexion extension and torsion of the torso and the lateral distance of the ball from the body at the instant of impact. This research involved a group of amateur players who performed a series of standardized gestures (forehands and serves) wearing a sensorized chest strap fitted with a wireless inertial unit. The collected data were processed to extract performance metrics. The percentage coefficient of variation for repeated measurements, Wilcoxon signed-rank test, and Spearman’s correlation were used to determine the system’s reliability. High reliability was found between sets of measurements in all of the investigated parameters. The statistical analysis showed moderate and strong correlations, suggesting possible applications in assessing and optimizing specific aspects of the technique, like the player’s distance to the ball in the forehand or the toss in the serve. The significant variations in technical execution among the subjects emphasized the need for tailored interventions through personalized feedback. Furthermore, the system allows for the highlighting of specific areas where intervention can be achieved in order to improve gesture execution. These results prompt us to consider this system’s effectiveness in developing an on-court mobile application.

## 1. Introduction

Tennis is a very traditional and popular sport, about which there have been written indications for around 500 years [1,2]. Being a discipline of very ancient origins, it has undergone mutations over time and has seen major changes due to recent technological developments in materials over the past three decades. For example, with more powerful and manageable racquets, ball speed has increased significantly, thus making it necessary to modify the biomechanics of fundamental strokes. Since the mid-1990s, service speed has increased substantially in both the first and second serves, allowing a significant increase in the number of aces and a change in the nature of the game [3]. Tennis is a highly technical sport that involves, in addition to large muscle groups, many small muscles necessary for the fine coordination of the athletic gesture [4,5].

For this reason, learning is particularly demanding; therefore, it is common to make various mistakes while improving skill levels [6]. One’s technique is an element that can be modified and refined continuously [7]. If improper actions are not corrected, they risk compromising the learner’s mastery of the complete technique or even result in injury [8]. Wrong actions can be corrected accurately and effectively with biomechanical analysis of movement.

Regarding physical mechanics, the perfect tennis technique effectively combines maximum strength and control while minimizing injury risk. Interesting insights have emerged from literature research on the ideal angles and body positions to maintain during the various phases of the shots, insights that were obtained while comparing high-level players with amateur players [5,9,10,11]. However, there are no practical, accessible, and low-invasive tools that can provide indications to tennis players and instructors on the most important parameters to train according to the level of play and characteristics of the athlete.

### 1.1. Game Fundamentals

The basic technical movements of tennis include serve, forehand, backhand, and volley. In modern tennis, where the play from the baseline predominates, the forehand is the most important technical action after the serve [12]. Studies have shown that, in the game, the forehand is more frequently played than the backhand and is often more effective as a last shot [12,13,14,15]. Tennis coaches and players constantly strive to improve strokes from a technical standpoint, particularly among the key factors of playing technique, i.e., coordination in using the racket and generating speed [16]. The main technical–tactical component of the game is power and accuracy. Indeed, the ability to hit the ball with great power is a defining characteristic of the modern game [17]. Younger players have yet to develop this ability, which, among others, could separate amateur athletes from high-performance athletes. However, developing power is not enough and needs to be undertaken with the correct timing, given that a difference of only one degree in the orientation angle of the string plate at impact can result in a difference of 40 cm in the placement of the ball in the opponent’s court [16,18]. In all of the shots, transferring the kinetic chain starts with the legs. In rebound shots, proper footwork allows the player to position themselves with an optimal stance and balance to transfer energy to the ball. The stances are classified into neutral (or closed), semi-open, and open. These may differ within the various tactical contexts so that one can speak of offensive or defensive open stances, for example. Additionally, there are also dynamic stance types. In the case of the forehand, a neutral stance is played with feet aligned perpendicular to the net, with the nondominant front foot in the open parallel to the net (feet aligned with each other), while intermediate stances are called semi-open. In any case, the nondominant foot generally has a stable support and fulcrum function in the forehand, and the limb mainly involved in pushing is the posterior one (i.e., the dominant one). The continuity and harmony of the movement can play a key role in the success of the stroke. Indeed, a harmonic structure has been found in the forehand of expert tennis players, who presented, independently of their own unique mannerisms, a similar temporal relationship between the main phases of the stroke, unlike amateur tennis players in whom this harmony was not found [19].

In this study, the execution techniques of the two most important game fundamentals, serve and forehand, were explored in detail.

The forehand is the shot made by hitting the ball, after rebound, from the right side of the body in right-handed players. The forehand is a continuous process in which we can observe different technical adaptations among tennis players; however, some technical parameters are present in each of them. The forehand can be divided into five main phases: starting position, preparation (unit-turn), opening phase, impact phase, and final [20]. The player begins the movement with a backward rotation of the hips and shoulders. The dominant upper limb (racket arm) proceeds backward with a semicircular opening, and they position themselves at the correct distance from the bounce of the ball, with a wide base of support and, depending on the game situation, with a “neutral,” “semi-open” or “open” stance [20]. In the impact phase, the racket moves forward with leg thrust and the rotation of the shoulders, trunk, and hips. When the racket approaches to touch the ball, leg thrust is important. Although the foot is off the ground, the body is still in the coordinated chain of power and speed transmission [21]. The impact occurs in front of the body, in varying positions and heights depending on the grip used and the playing situations. When the ball is touched, the momentum of the ball and the distance of the ball from the center of the racket produce a rotational torque, causing the velocity generated by the racket head as an angular movement of the trunk to be transferred to the arm and wrist to enable the proper transfer of the kinetic chain [9,16,22]. The racquet proceeds forward overhead until it, usually, ends above the nondominant shoulder [20].

In the forehand, the maximum internal rotation velocity was found to occur quite late in the forward swing phase [23,24]. During the entire movement, the trunk, head, and shoulders form the basic elements for maintaining balance, and they must coordinate to ensure the shot’s accuracy [9].

In rebound shots (forehands and backhands), however, the execution technique is strongly influenced by the tactical context and foot stance [25,26,27]. In addition to significant differences in the technique of shots played in open (or semi-open) versus neutral stances, the distance of the ball conditions the technical gesture, for which defensive situations are adapted to the tactical context [7]. However, when given sufficient time to prepare the shot, an expert tennis player’s contact with the ball occurs at approximately the same lateral distance from the body [28].

The serve is the first shot played at each point and is undoubtedly the most important shot in the game. With an effective serve, the player can directly win the point (with an ace, or a winning serve) or gain a tactical advantage over the opponent. It is the only shot in tennis that is not played in displacement—is not directly conditioned by the opponent—and for which the tennis player decides where to impact the ball. At the same time, it is one of the most complex and challenging to learn shots, requiring different coordination skills [29,30,31,32,33]. Indeed, a well-executed serve results in a coordinated action of all body segments in a kinetic chain from the lower to the upper limbs [34,35]. Serving can be split into six phases: preparation, ball toss and loading, trophy position, acceleration phase, impact, and follow-through [36,37]. During the loading phase, the dominant arm, referred to in tennis jargon as the “racquet arm,” rises directly or usually follows a semicircular trajectory toward the trophy position. At the end of the loading phase, which has various personalities, all players reach the position called the “trophy position” in tennis jargon. In this position, the athlete has reached maximum loading of the lower limbs, hips, and trunk, the racket tip looks upward, and the arm-throw is extended overhead [20]. The player generates power through trunk torsion and shoulder extension, essential for maximizing racquet speed at ball impact [33,38]. The dominant shoulder, first low and below the level of the other, flips up (shoulder-over-shoulder), with the thrust of the legs and the racket directed to impact the ball high. After the impact, the racquet proceeds forward to end below the nondominant arm, maintaining the “arabesque” position [20].

Each phase requires careful integration of strength and coordination [32]. One of the phases that most affect stroke success, particularly in amateur tennis, is the ball toss [32,37,39]. Inaccurate tosses can compromise the coordination of the entire action, whereas an accurate toss allows for proper timing between the loading and acceleration phases. Proper kinetic chain activation is critical for effective service, allowing energy to be transferred from the legs to the trunk and, finally, to the arm, minimizing stress on the joints [40,41]. In recent years, biomechanical research has increasingly focused on preventing service-related injuries [42,43]. Studies have shown that incorrect serving techniques can increase the risk of conditions such as, for example, rotator cuff tendinopathy and tennis elbow syndrome [44,45], underscoring the importance of detailed biomechanical analysis by which to optimize performance and preserve the athlete’s health.

### 1.2. Assessment Systems for Game Fundamentals

Two-dimensional and three-dimensional video analyses are widely used systems for technical evaluation through marker-based or markerless systems [46,47,48,49,50,51,52,53,54,55,56] in sports. Measuring and evaluating athletes’ technical movements can help them improve their technical level and correct and optimize special movements to prevent injuries and improve sports performance. As a valid alternative to optical motion tracking systems, inertial sensors (inertial motion units (IMUs)) have been proposed for the kinematic analysis of strokes in tennis [57,58,59]. Usually, IMUs contain MEMS-type gyroscopes, accelerometers, and magnetometers, and, through a sensor fusion estimation framework, 3D orientation is obtained. Despite their small size and applicability in almost any environment, these devices face several technical challenges [60]. For instance, IMU gyroscopes are valid alternatives to 3D optical motion capture systems for angular kinematics analysis in tennis [61]. In other cases, inertial sensors have been placed on the racket or wristband for stroke analysis and classification [62,63]. Although this approach allows for the direct measurement of important kinematic parameters, such as accelerations, angular velocities, and the exact position of joint segments using quaternions and global reference systems, the extensive use of these devices may prove invasive. Although very lightweight, several sensors placed on the tennis player’s body could sensitively limit movement or otherwise impair the naturalness of the gesture. In the same way, even a single sensor of a few grams placed on the tennis racket can compromise the technique of the strokes. The advantages of inertial sensors include, among others, the accuracy, convenience, and quickness of measuring and analyzing data, which can potentially take place even in real time without the need for very expensive hardware components, unlike current 3D motion capture systems [47]. At present, most of the literature focuses on researching the upper limb kinematic characteristics of forehand, backhand and service movements in tennis [49,51,64]; however, practical court tools are still limited, so coaches rely mainly on experience to teach and train technical fundamentals.

An easy-to-use, affordable, and minimally invasive practical court application would make it easier for coaches to assess technique and give feedback, particularly to young tennis players and beginners who have not yet acquired sufficient awareness of the technical gesture. In previous preliminary studies, by applying an inertial sensor, a strong correlation was found between the torso tilt detected by the IMU system and the lateral distance to the ball at the impact point in the forehand of three amateur tennis players [65,66].

In light of these considerations, this study aims to establish the level of reliability of this single inertial sensor system applied to the assessment of forehand and serve technique in amateur players. Among the technical parameters, in addition to a confirmation of what has already been highlighted, we expect a strong correlation between trunk angles (tilt and rotation) and lateral distance of the ball not only in the forehand but also in the serve. This is assumed to allow the system to be used on the court to provide live feedback to players on refining the search for proper distance and toss.

## 2. Materials and Methods

### 2.1. Data Collection

This study used two data acquisition protocols that differ for equipment setup and camera positioning: the first aimed at analyzing the forehand and the latter for the serve. All of the measurements were conducted on different days between June 2023 and July 2024 in outdoor courts (hard-surface and clay courts) with no adverse weather and negligible wind conditions (less than 4 km/h, according to measurements from the Rome Ciampino meteorological station). The study involved a total of 34 amateur (players of various skill levels, with irregular or occasional weekly sports practice, non-professionals) tennis players (*n* = 34: 13 females, 21 males) from 16 to 60 years old, of different stature, and from four different tennis clubs. The players had an average of 5 years of playing experience (without stop periods longer than a few weeks). All recruited subjects were in good health, had no recent injuries, and gave their consent to data processing for research purposes. In both protocols, the subjects wore specially prepared elastic chest straps with a holder, inside which an Xsens Movella DOT inertial sensor was placed [60,67,68]. This was positioned as tightly as possible, without allowing free movement to the sensor, and as comfortably as possible for each subject, depending on their physical conformations. The previously calibrated inertial sensors were set to a 120 Hz offline dynamic acquisition mode. Written informed consent was obtained from participants before being tested. The research was approved by the Internal Research Board of “Tor Vergata” University of Rome. All procedures were carried out in accordance with the Declaration of Helsinki.

### 2.2. Forehand Assessment

**Figure 1:**
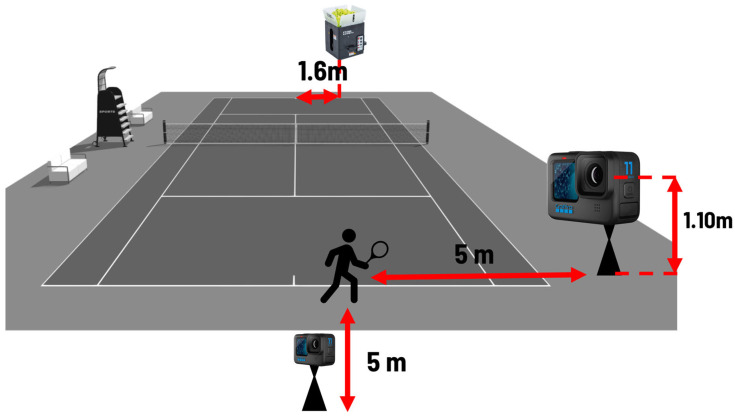
Illustration of the setup for forehand measurements: the two action cameras were aligned about 5 m from the point of impact and placed on a tripod at 1.10 m above the ground; the Tennis Tutor Plus ball-launching machine was positioned on the ground near the opposite baseline at 1.60 m from the mid-point.

The study of tennis forehand involved 21 amateur players (8 females, 13 males; age: 39 ± 13 years; height: 173 ± 9 cm; experience mean 5.10 years). Following a 20 min physical and technical warmup, all subjects in the examined sample played two sets of 10 forehand strokes in a controlled situation, with a three-minute rest between sets. A Tennis Tutor Plus ball-launching machine, positioned on the opposite baseline at 1.60 m from the mid-point, was set up to provide easy-to-handle balls. (Figure 1).

**Figure 2:**
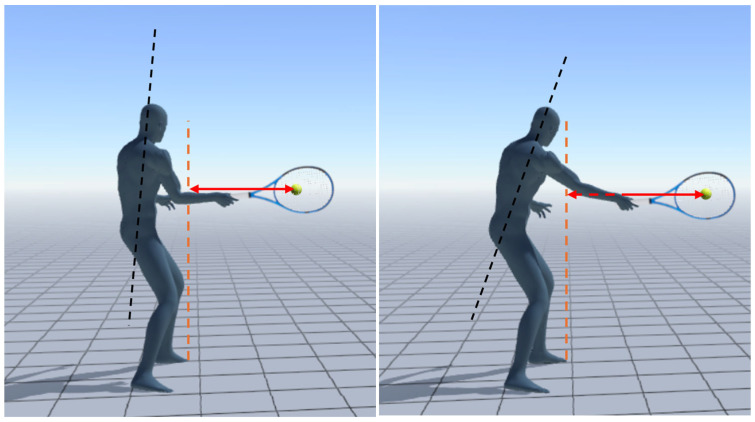
Illustration of different body positions and trunk inclination (black dashed line) in relation to the ball distance from the longitudinal axis (orange dashed line), coincident with the first toe of the nondominant foot. The arrows represent the distance between the ball and the longitudinal reference axis.

Two action cameras at 240 Hz captured the twenty forehands of each player from lateral and rear perspectives, aligned about 5 m from the point of impact, and placed on a tripod at 1.10 m above the ground. The cameras were synchronized with each other through a light pulse. Video analysis was conducted to identify the lateral distance of the ball at the point of impact from the longitudinal axis, coincident with the first toe of the nondominant foot (Figure 2). The procedures and calibration system are detailed in Section 2.4. In the stroke analysis, kinematic parameters of acceleration, velocity, and angular velocity recorded by the IMU were used as performance parameters, while lateral distance, stance type, stroke direction, and torso angle and orientation (explained in Section 2.5) were used as technical parameters.

### 2.3. Serve Assessment

Thirteen amateur tennis players (4 females, 9 males; age: 35 ± 15 years; height: 173 ± 10 cm; experience: 6.23 years) played two sets of 5 flat serves each from the deuce side after a 20 min physical and technical warmup. Two action cameras at 240 Hz captured the ten services of each player from lateral and rear perspectives, aligned about 5 m from the point of impact and placed on a tripod at 1.55 m above the ground.

**Figure 3:**
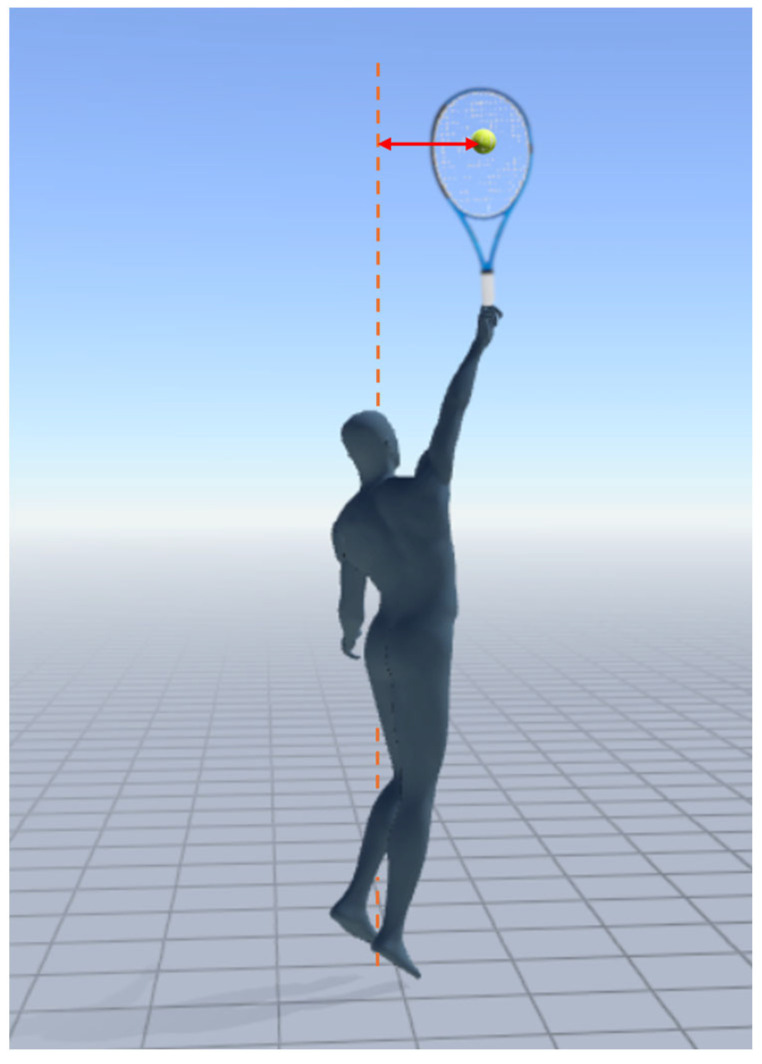
Ball distance detection during serve at the instant of impact from the longitudinal axis, coincident with the first toe of the nondominant foot in the starting position. The arrow represents the distance between the ball and the longitudinal reference axis.

The cameras were synchronized with each other through a light pulse. Video analysis was conducted to identify the anterior–posterior (APD) and lateral distance of the ball at the point of impact from the longitudinal axis coincident with the first toe of the nondominant foot in the starting position, placed near a target appositely placed on the ground (Figure 3). The procedures and calibration system are detailed in Section 2.4.

In each impact frame, calibration was re-performed using the racquet of a known size, equivalent to 68.50 cm, which was used as a reference and placed on the measurement plane. In the service analysis, the kinematic parameters of acceleration, velocity, and angular velocities recorded by the IMU were used as performance parameters, while lateral and anteroposterior distance and torso angle (explained in Section 2.5) were used as technical parameters.

### 2.4. Video Analysis

**Figure 4:**
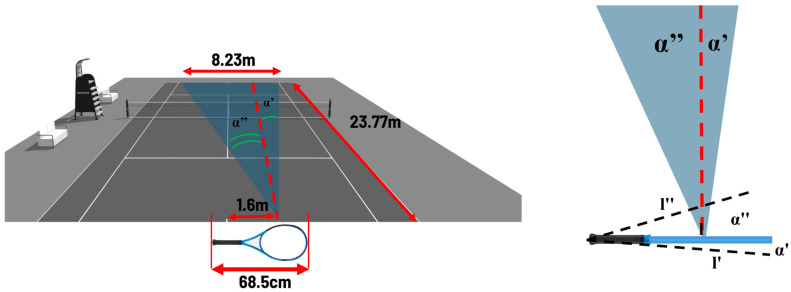
Illustration of the change in length of the reference object (the racquet) on the camera plane due to the tilt of the racquet. The arrows represent the width (8.23 m) and length (23.77) of the tennis court, and the length of the racket (68.5 cm) positioned during impact about 1.6 m from the center of the court. Angles α′ and α″ constitute the maximum inclination of the racket with respect to perpendicularity to the dashed red line, and consequently l′ and l″ the maximum possible deformation in length of the tool observed from the rear camera.

Video analysis was conducted using Kinovea (Version 0.9.5) [69] software. The toe of the nondominant foot was chosen as the reference point in both gestures because it is positioned in approximately the same plane for impacts of each of the different stance techniques and therefore represents, in the structured situations of this test, the fixed point of support on the ground during the execution of the forehand. The calibration was conducted using the racquet size as a reference placed on the measurement plane. Although the test recreated a controlled situation, in the forehand assessment, players naturally did not have the same position on the court in each shot. For this reason, in each impact frame, calibration was re-performed using the racquet length, equivalent to 68.50 cm, as a reference placed on the measurement plane (all subjects used standard-length racquet models). The inclination of the racquet during the impact, which was included at an angle α of about 20° (α″ ≈ 13.7°; α′ ≈ 6.4°), could affect the accuracy of the calibration process with an error of less than 1% for the longline shots and within 3% for cross shots (Figure 4). In fact, in the case of longline shots, the angle α′ implies a maximum measurement error on the camera plane of the racquet of about 0.5 cm less than the actual size (l′ ≈ 68.07 cm; l = 68.5 cm; Δ_max_ ≈ 0.5 cm). In cross shots, the α″ angle results in a maximum difference, Δ_max_, of about 2 cm (l″ ≈ 66.55; l = 68.5 cm). In the cross shots, therefore, 1 cm was subtracted from the length of the calibration object so that the error was halved.

### 2.5. Inertial System Data Analysis

**Figure 5:**
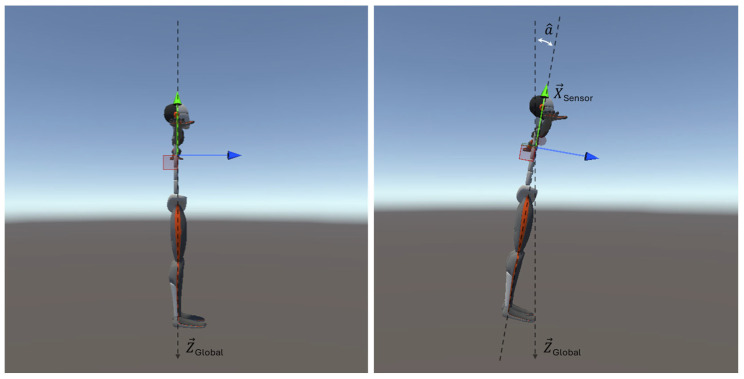
Illustration of the angle of inclination of the trunk (α) between the *X*-axis of the sensor and the global *Z*-axis (the direction of the Earth’s gravitational force). The dashed black lines represent the direction of the Earth’s gravitational force ($\vec{Z}$ Global) and the $\vec{X}$ axis of the sensor (coincident with the green arrow); while the blue arrow represents the $\vec{Z}$ axis of the sensor.

From the data acquired, the trunk tilt angle ($\hat{\alpha })$, i.e., the complementary Euler Y-angle, corresponding to the angle between the *X*-axis of the sensor and the global *Z*-axis (the direction of the Earth’s gravitational force), was computed using quaternions math (Figure 5).

**Figure 6:**
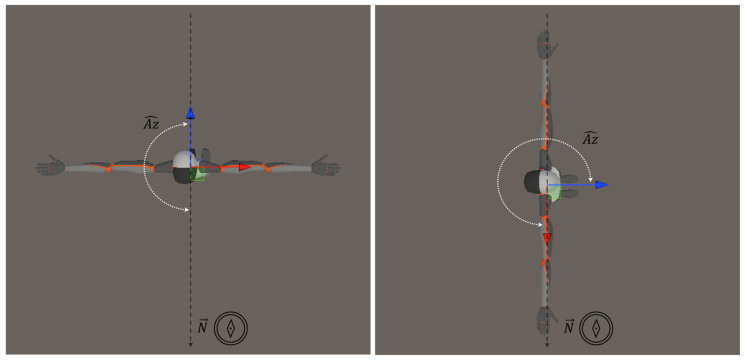
Illustration of the trunk rotation angle $\left(\hat{Az}\right)$ on the horizontal plane with respect to the direction of the Earth’s magnetic north, $\vec{N}$. The black dashed line represents the direction of Earth’s magnetic north ($\vec{N}$), the blue and red arrow stand for the $\vec{Z}$ and $\vec{Y}$ axis of the sensor, respectively, while the white dashed line marks the azimuth angle ($\hat{Az}$).

In addition, the azimuth $(\hat{Az}),$ the rotation angle on the horizontal plane with respect to the direction of the Earth’s magnetic north was calculated using magnetometer data (Figure 6).

Although the orientation of the courts in which the measurements were taken was approximately the same with respect to magnetic north, an IMU sensor calibration was performed. For this purpose, the subject was measured in an orthostatic position, with both feet placed near the baseline and facing the playing field. In the forehand assessment, data of acceleration (Acc Z), horizontal velocity (dv), angular velocity (Gyr X), trunk inclination angle ($\hat{\alpha })$, and azimuth $\left(\hat{Az}\right)$ during the impact phase (coinciding with the point of maximum angular velocity of trunk rotation [23]) were analyzed. These were also investigated according to different stance techniques and ball directions. In the serve assessment data of acceleration (Acc Z and Acc X), torsion angular velocity (Gyr X), shoulder-over-shoulder angular velocity (Gyr Z), and trunk inclination angle ($\hat{\alpha })$ during the impact phase were analyzed.

### 2.6. Statistical Analysis

The Shapiro–Wilk test was used to validate the assumption of normality. Because it was found that the analyzed data did not follow the normal distribution, nonparametric tests were used for inferences. In the forehand, the intraclass correlation coefficient (ICC) [70], the percentage coefficient of variation (CV%) for repeated measurements, and Spearman’s correlation (ρ) were calculated to determine the reliability between the two sets of measures for the lateral distance (LD), torsion angular velocity (Gyr X), horizontal acceleration (Acc Z) and velocity (dv), the trunk angles ($\hat{\alpha }$) and azimuth $\left(\hat{Az}\right)$. In the serve assessment, ICC, CV%, and Spearman’s correlation were calculated to determine the reliability between the two sets of measures for the LD and anteroposterior distance (APD), torsion angular velocity (Gyr X), shoulder-over-shoulder angular velocity (Gyr Z), horizontal acceleration (Acc Z), vertical acceleration (Acc X) and the trunk angle ($\hat{\alpha }$). In addition, in both protocols for the investigated parameters, standard error of measurement (SEM) [71] and minimal detectable change (MDC) based on a 95% confidence level were calculated using bootstrapping. Moreover, Spearman’s correlation was used as an assessment test of consistency and the of repeatability of quantitative measurements made by the same operator in the video analysis of the two sets. Wilcoxon signed-rank *t*-test and Spearman’s correlation coefficient were used for comparisons between groups, for repeatability of test measures, and to determine the level of specificity among selected test variables. Effect sizes (ESs) given by the rank-biserial correlation were calculated between first and second set averages [72], where a small ES was 0.10–0.29, moderate 0.30–0.49, and large >0.50 [73,74]. Bland–Altman plots with 95% limits of agreement (LoA) were used to show the within-subject variation and systematic differences between the two session trials. In addition, Spearman’s correlation between lateral ball distance and trunk inclination, azimuth, angular velocity of trunk rotation, and horizontal component of acceleration and velocity were investigated for each player and for the whole group. The best forehand and service corresponding to shots in which subjects expressed a higher than eighty percentile value of acceleration (Acc Z) and angular velocity (Gyr X) were also studied. Statistical analysis of the data was carried out with MedCalc software (Version 23.0.2) [75] and R software (Version 4.4.2) [76].

### 2.7. Exclusion Criteria

Data from 21 subjects in the forehand protocol and 13 in the serve were considered. In the forehand, shots played in open and closed stances were also investigated separately. Players who had made at least three trials with the investigated stance were considered in the stance analysis. Semi-open stances were not examined in detail because of the low amount of data. Shots played in open stance by 11 players and in neutral stance by 15 players were then analyzed in detail.

## 3. Results

### 3.1. Forehand

In the analysis of the forehand stroke, 420 total shots were analyzed, including shots played in open, semi-open and neutral stances and at the cross, center, and longline.

#### 3.1.1. Reliability

Test–retest values of mean, SD, median, inter-quartile range (IQR), Spearman’s correlation (ρ), CV%, Wilcoxon signed-rank test, and the ES relative to the lateral distance (LD), torsion angular velocity (Gyr X), horizontal acceleration (Acc Z) and velocity (dv), trunk angles ($\hat{\alpha }$) and azimuth ($\hat{Az}$) performed in the two sets of measurements are reported in Table 1.

Wilcoxon signed-rank *t*-test for paired samples found no significant differences between the first and second sets of measurements, and the effect size is small or moderate in the case of the angle $\hat{Az}$. The two sets of measurements have a significantly strong Spearman correlation and medium in the $\hat{\alpha }$ angle.

ICC was more than 0.95 in all parameters except in the angle $\hat{\alpha }$. CV% was high in all parameters and particularly high in the angle $\hat{\alpha }$. SEM and MDC were low in Acc Z and dv, while higher values were found in the others (Table 2).

**Figure 7:**
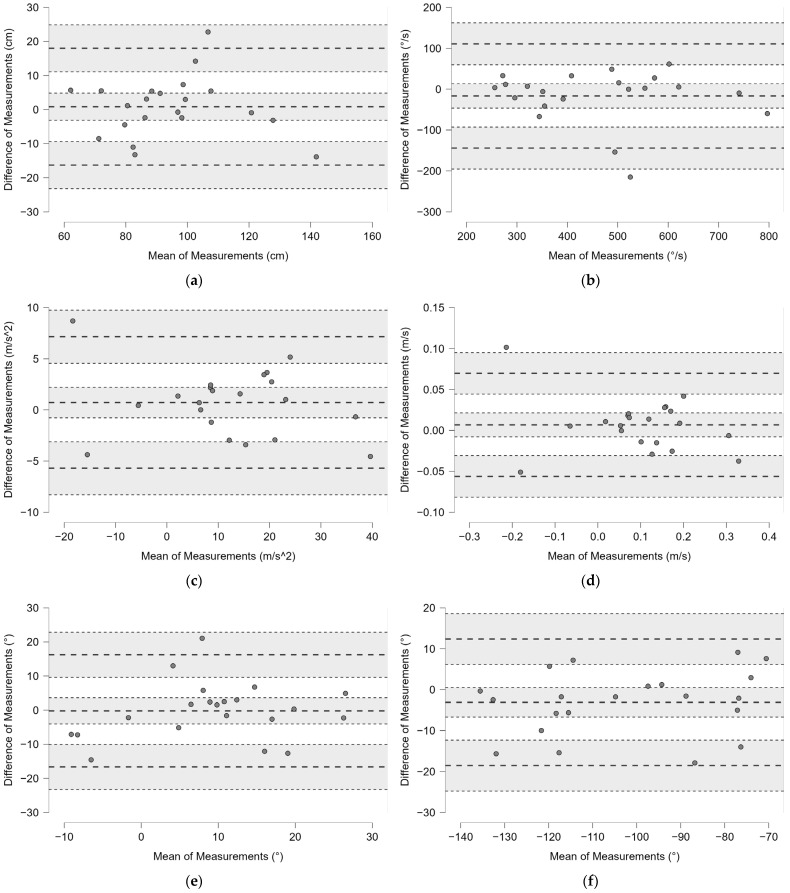
Bland–Altman plots with 95% limits of agreement (LoA) showing the difference of measurement between the two session trials relative to lateral distance (**a**); Gyr X (**b**); Acc Z (**c**); dv (**d**); $\hat{\alpha }$ (**e**); and $\hat{Az}$ (**f**). The bold dashed lines represent the mean difference and the limits (LoA), while the dotted lines and the gray background show the 95% confidence intervals.

Bland–Altman plots with 95% limits of agreement (LoA) have shown the difference of measurement between the two session trials relative to lateral distance (Figure 7a); Gyr X (Figure 7b); Acc Z (Figure 7c); dv (Figure 7d); $\hat{\alpha }$ (Figure 7e); and $\hat{Az}$ (Figure 7f).

#### 3.1.2. Data Variability

**Figure 8:**
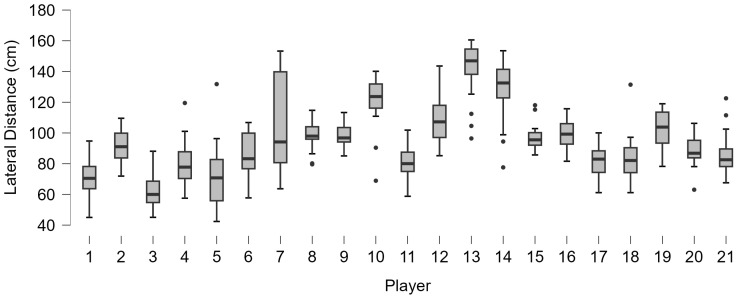
Boxplot distribution of the lateral distance of all of the shots played by the 21 players. The dots indicate the outliers.

**Figure 9:**
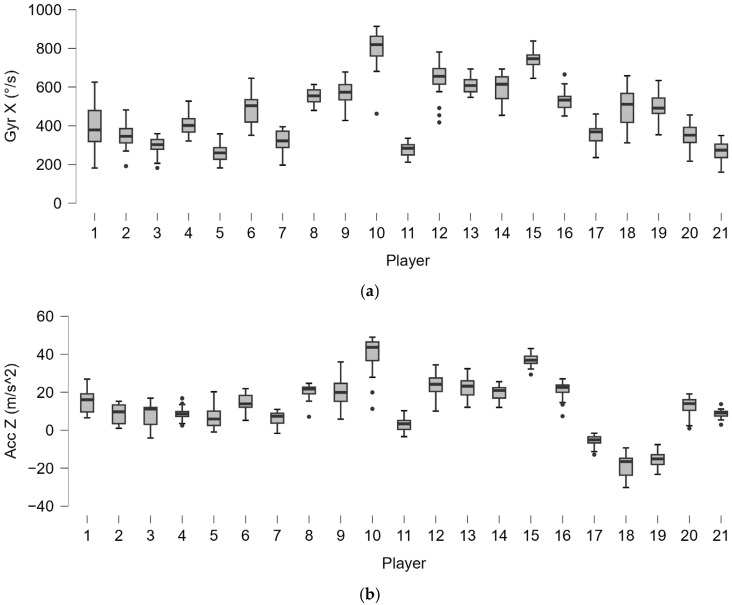
Boxplot distribution of the angular torsion velocity (Gyr X) (**a**) and horizontal acceleration (Acc Z) (**b**) of all of the shots played by the 21 players. The dots indicate the outliers.

**Figure 10:**
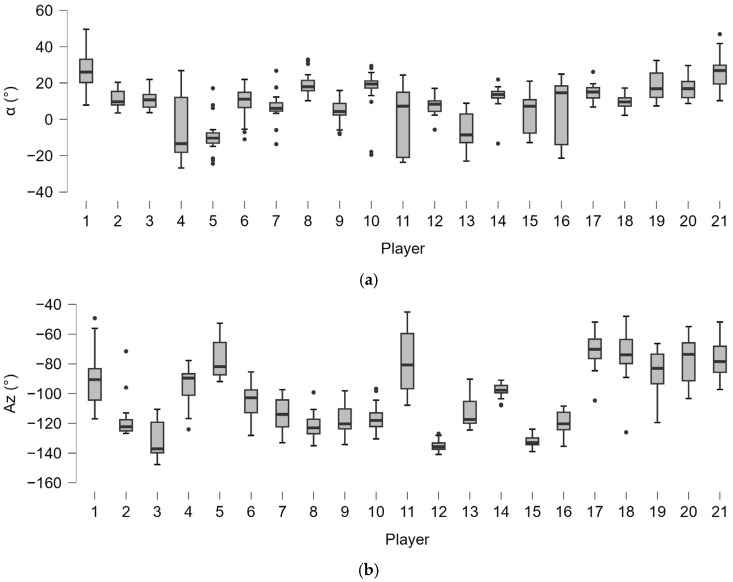
Boxplot distribution of trunk angles $\hat{(\alpha })$ (**a**) and azimuth ($\hat{Az}$) (**b**) of all of the shots played by the 21 players. The dots indicate the outliers.

The forehand analysis shows different distributions of lateral distance from the ball in players (Figure 8). As well as for lateral distance, the distribution of kinematic motion parameters (Figure 9) and trunk angles (Figure 10) shows wide subjective variability.

**Figure 11:**
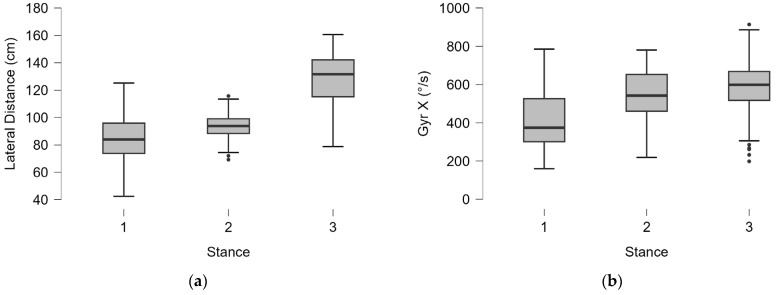
Correlation between stance type (1: neutral stance; 2: semi-open stance; 3: open stance) and lateral distance (**a**) and angular torsion velocity (Gyr X) (**b**). The dots indicate the outliers.

Moderate statistically significant correlations were found between the type of stance used by the player and lateral distance (ρ = 0.634, *p* < 0.001) and Gyr X (ρ = 0.454, *p* < 0.001), showing higher average and maximal velocities achieved in open stances (Figure 11).

**Figure 12:**
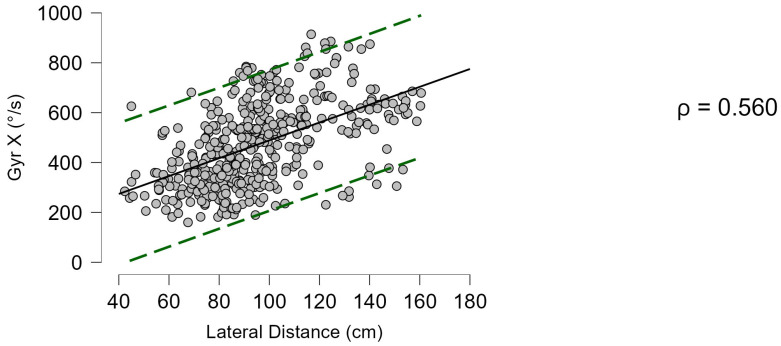
Correlation between lateral distance and angular torsion velocity (Gyr X). Green dashed lines indicate 95% predictions intervals.

More moderate correlations were found between the investigated parameters and stroke direction for lateral distance (ρ = 0.329, *p* < 0.001) and Gyr X (ρ = 0.251, *p* < 0.001). A medium correlation was found between lateral distance and Gyr X (ρ = 0.560, *p* < 0.001) (Figure 12). Minor but still statistically significant correlation indices were found between distance and direction (r = 0.333, *p* < 0.001), years of experience (r = 0.251, *p* < 0.001), and height (r = 0.131, *p* = 0.006).

Of all of the shots played, 276 forehands were played in a neutral stance (62.44%), 97 in an open stance (21.95%), and 69 in a semi-open stance (15.61%). Shots played in neutral and open stances were analyzed in detail.

#### 3.1.3. Neutral Stance

**Figure 13:**
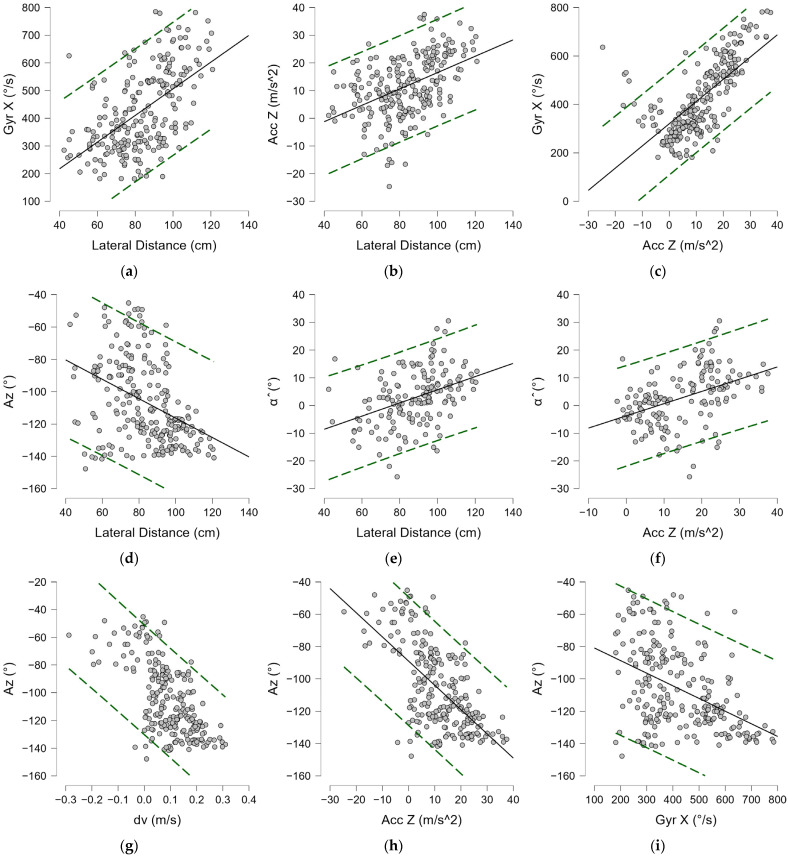
Spearman’s correlation (ρ) in the neutral stance forehand: (**a**) Gyr X—Lateral distance; (**b**) Acc Z—Lateral distance; (**c**) Gyr X—Acc Z; (**d**) $\hat{Az}$—Lateral distance; (**e**) $\hat{\alpha }$—Lateral distance; (**f**) $\hat{\alpha }$—Acc Z; (**g**) $\hat{Az}$—dv; (**h**) $\hat{Az}$—Acc Z; (**i**) $\hat{Az}$—Gyr X. Lateral distance; Gyr X: trunk angular torsion velocity; Acc Z: horizontal acceleration; dv: horizontal velocity; $\hat{\alpha }$: trunk angle; $\hat{Az}$: azimuth. Green dashed lines indicate 95% predictions intervals.

Among the shots played in the neutral stance, i.e., the stance with the least technical variance in support, there is considerable variation among each player of the technical parameters (20% in the lateral distance, 35% in the Gyr X, 91% in the Acc Z). The average lateral distance detected in the players examined was 84.73 ± 16.39 cm, with a minimum of 42.37 cm and a maximum of 122.55 cm. Within the whole sample, a moderate positive correlation index was found between torso frontal tilt angle $\hat{\alpha }$ and lateral distance to the ball (ρ = 0.432, *p* < 0.001) (Figure 13e) and a negative one was found between $\hat{Az}$ and distance to the ball (ρ = −0.402, *p* < 0.001) (Figure 13d). A strong correlation was found between torsion angular velocity and acceleration along the Z-axis (ρ = 0.732, *p* < 0.001) (Figure 13c). Positive correlation indices were found between lateral distance and Gyr X (ρ = 0.568, *p* < 0.001) and Acc Z (ρ = 0.487, *p* < 0.001), shown in Figure 13a and Figure 13b, respectively. Negative correlations were found between the angle $\hat{Az}$ and all performance parameters, as shown in Table 3.

In neutral stance forehands, the best observed performance in terms of acceleration and angular velocity (higher than 80 percentile) occurred at the ball distances in the range of −0.29 to +2.19 standard dev., between 79.26 and 120.31 cm. The optimal torso angles at which the best performance was expressed were between 1 and 30 degrees ($\hat{\alpha })$ with respect to frontal tilt and in a range of ±10 degrees in the orientation of the shoulder line with respect to the net ($\hat{Az}$ − 130 ± 10 degrees).

#### 3.1.4. Open Stance

**Figure 14:**
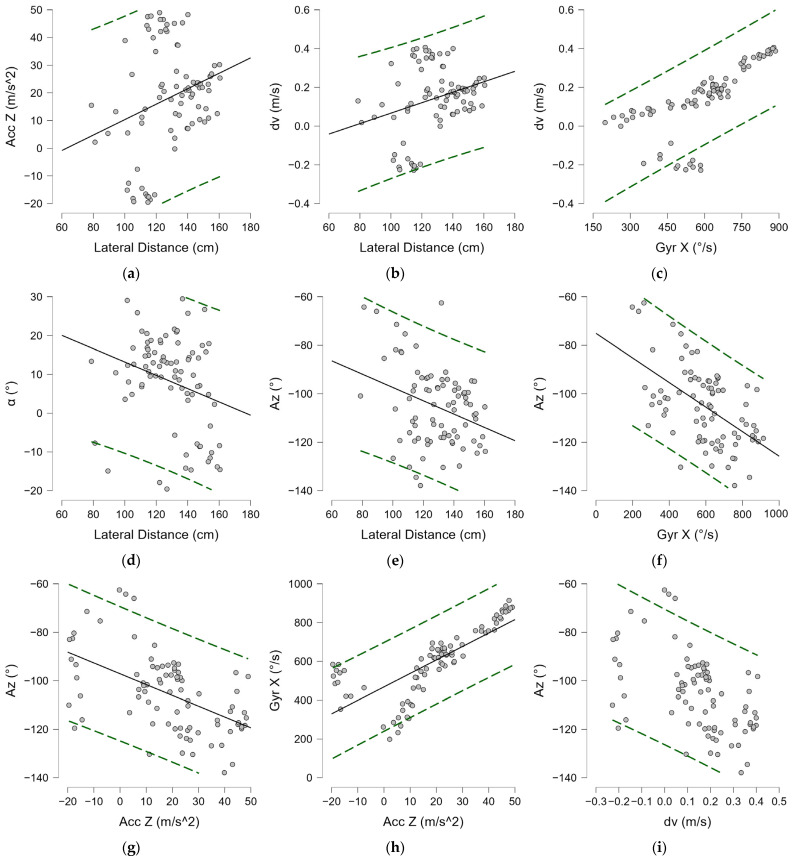
Spearman’s correlation (ρ) in the open stance forehand: (**a**) Acc Z—Lateral distance; (**b**) dv—Lateral distance; (**c**) dv—Gyr X; (**d**) $\hat{\alpha }$—Lateral distance; (**e**) $\hat{Az}$—Lateral distance; (**f**) $\hat{Az}$—Gyr X; (**g**) $\hat{Az}$—Acc Z; (**h**) Gyr X—Acc Z; (**i**) $\hat{Az}$—dv. Lateral distance; Gyr X: trunk angular torsion velocity; Acc Z: horizontal acceleration; dv: horizontal velocity; $\hat{\alpha }$: trunk angle; $\hat{Az}$: azimuth. Green dashed lines indicate 95% predictions intervals.

Among forehands played in the open stance, a mean lateral distance from the nondominant foot of 128.71 ± 18.83 cm was found, with a variance of 14.6%, a minimum of 78.77 cm and a maximum of 160.55 cm. Modest negative correlations were found between lateral distance and frontal torso angle $\hat{\alpha }$ and $\hat{Az}$, shown in Figure 14d and Figure 14e, respectively, and reported in Table 4.

Positive correlations were found between lateral distance and horizontal velocity and acceleration; a strong positive correlation was found between Gyr X, velocity, and acceleration, as shown in Figure 14b and Figure 14a, respectively, and reported in Table 4. Negative correlations are also present between $\hat{Az}$, and all performance parameters (dv, Acc Z, and Gyr X) (Table 4). The best observed performance in terms of acceleration and angular velocity occurred at the ball distances in the range of −0.81 and +0.61 with standard deviation from the mean, or between 114 and 140 cm. The optimal torso angles at which the best performance was expressed were between 9 and 29 degrees $\hat{\alpha }$ with respect to frontal tilt and in a range of ±10 degrees in the orientation of the shoulder line with respect to the net ($\hat{Az}$ − 130 ± 10 degrees).

### 3.2. Serve

In the analysis of the stroke of the serve, 130 total shots were analyzed, regardless of the direction and result of the shot (inside or outside the service area). The type of leg loading in the service was not considered.

#### 3.2.1. Reliability

Test–retest values of mean, SD, median, inter-quartile range (IQR), Spearman’s correlation (ρ), Wilcoxon signed-rank test and the rank-biserial correlation relative to the lateral and anterior–posterior distances, torsion angular velocity (Gyr X), shoulder-over-shoulder velocity (Gyr Z), horizontal acceleration (Acc Z), vertical acceleration (Acc X) and velocity (dv), trunk angles ($\hat{\alpha }$) and azimuth ($\hat{Az}$) performed in the two sets of measurements are reported in Table 5. Wilcoxon signed-rank *t*-test for paired samples found no significant differences between the first and second sets of measurements, and the effect size was small or moderate in ADP and $\hat{\alpha }$ or large in the case of LD and Gyr Z. The two sets of measurements have a significantly strong Spearman correlation.

ICC was higher than 0.95 in all parameters. CV% was high in all parameters and extremely high in the lateral distance shown in Table 6. SEM and MDC were low in Acc X, while higher values were found in the other parameters (Table 6).

**Figure 15:**
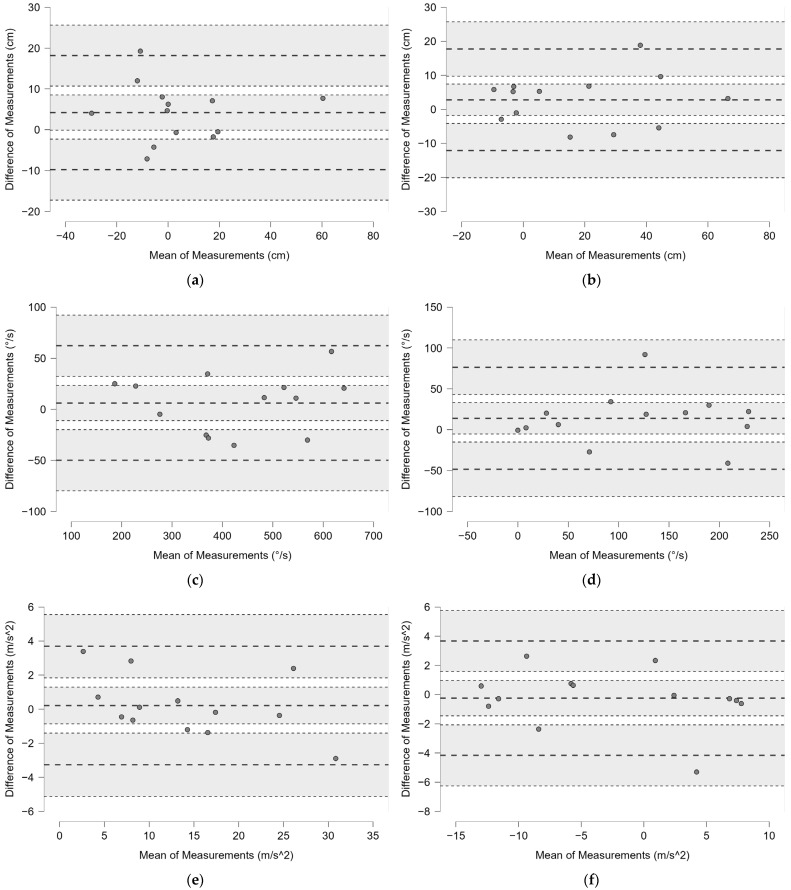
Bland–Altman plots with 95% limits of agreement (LoA) showing the difference of measurement between the two session trials relative to lateral distance (**a**); APD (**b**); Gyr X (**c**); Gyr Z (**d**); Acc Z (**e**); Acc X (**f**). The bold dashed lines represent the mean difference and the limits (LoA), while the dotted lines and the gray background show the 95% confidence intervals.

**Figure 16:**
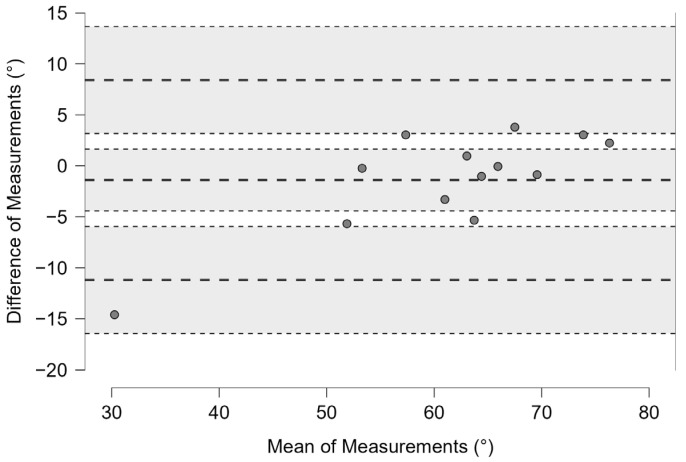
Bland–Altman plot with 95% limits of agreement (LoA) showing the difference of measurement between the two session trials relative to the angle $\hat{\alpha }.$ The bold dashed lines represent the mean difference and the limits (LoA), while the dotted lines and the gray background show the 95% confidence intervals.

Bland–Altman plots with 95% limits of agreement (LoA) have shown the difference of measurement between the two session trials relative to lateral distance (Figure 15a); APD (Figure 15b); Gyr X (Figure 15c); GyrZ (Figure 15d); Acc Z (Figure 15e); Acc X (Figure 15f); $\hat{\alpha }$ (Figure 16).

#### 3.2.2. Data Variability

**Figure 17:**
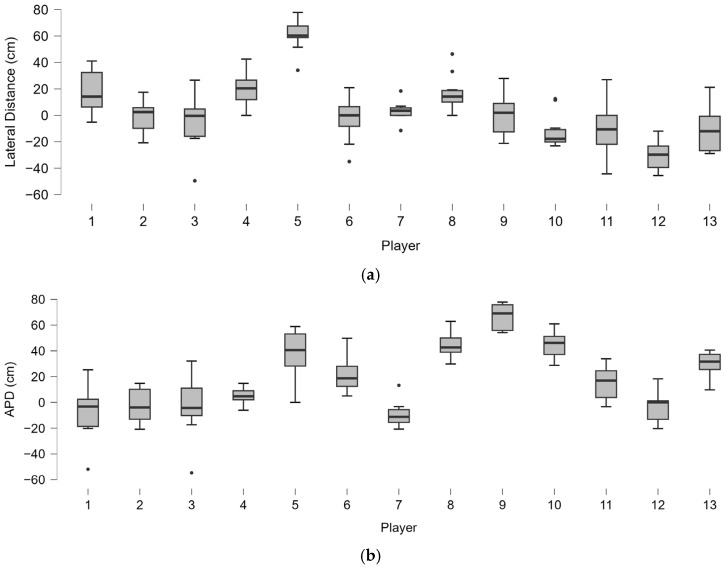
Boxplot distribution of the lateral (**a**) and anteroposterior (**b**) distance in the serves played by the 13 players. The dots indicate the outliers.

In the serve assessment, different distributions of lateral (Figure 17a) and anteroposterior distance (APD) from the ball were found in the players (Figure 17b).

**Figure 18:**
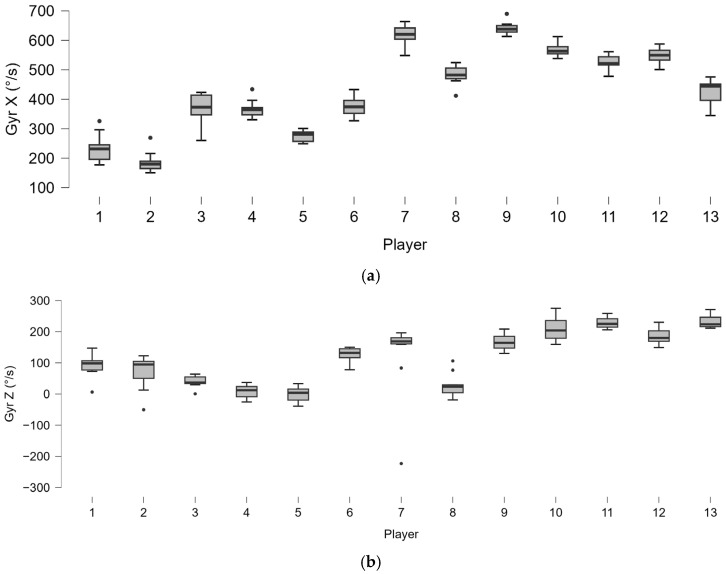
Boxplot distribution of the torsion angular velocity (Gyr X) (**a**) and shoulder-over-shoulder angular velocity (Gyr Z) (**b**) in the serves played by the 13 players. The dots indicate the outliers.

A mean lateral distance of 3.76 ± 25 cm and an anteroposterior (APD) distance of 18.35 ± 27 cm was found in the whole group. In the former case, a minimum was found of −49.56 cm and a maximum of 77.86 cm, and, in the ADP, minimum and maximum values corresponded to −54.73 cm and 77.91 cm, respectively. An average torso tilt angle $\hat{\alpha }$ of 28.60 ± 12 cm, an average torsion speed of 430.85°/s (Figure 18a), and an angular shoulder-over-shoulder angular speed of 116.36 ± 91°/s (Figure 18b) were found.

#### 3.2.3. Inferential Statistics

**Figure 19:**
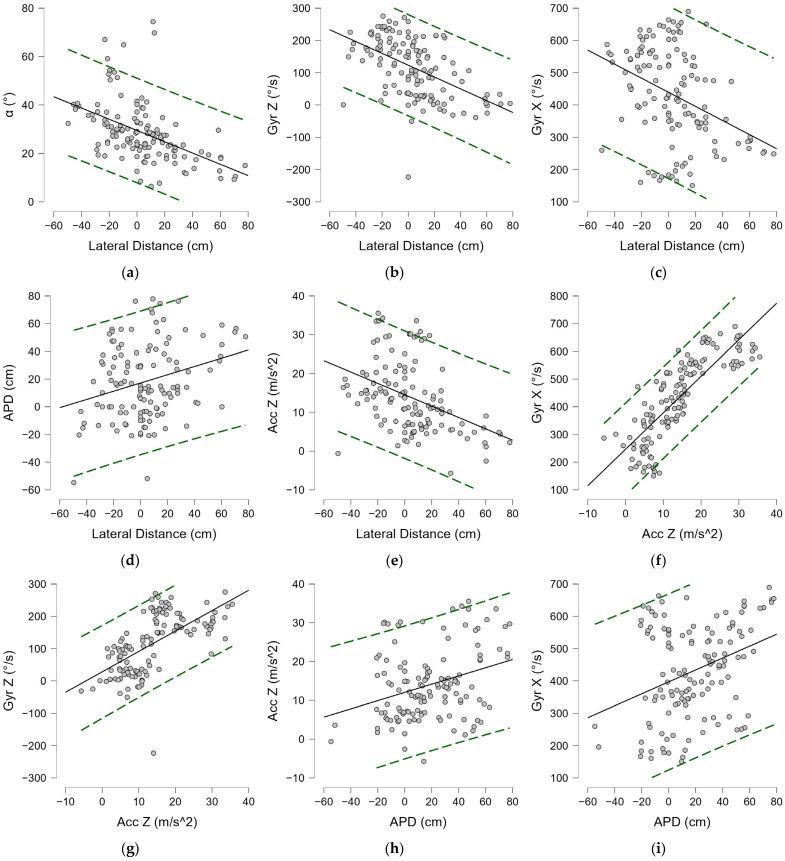
Spearman’s correlation (ρ) in the serve: (**a**) $\hat{\alpha }$—Lateral distance; (**b**) Gyr Z—Lateral distance; (**c**) Gyr X—Lateral Distance; (**d**) ADP—Lateral distance; (**e**) Acc Z—Lateral distance; (**f**) Gyr X—Acc Z; (**g**) Gyr Z—Acc Z; (**h**) Acc Z—ADP; (**i**) Gyr X- ADP. Lateral distance; Gyr X: trunk angular torsion velocity; Gyr Z: shoulder-over-shoulder angular velocity; Acc Z: horizontal acceleration; ADP anterior–posterior distance; $\hat{\alpha }$: trunk angle. Green dashed lines indicate 95% predictions intervals.

**Figure 20:**
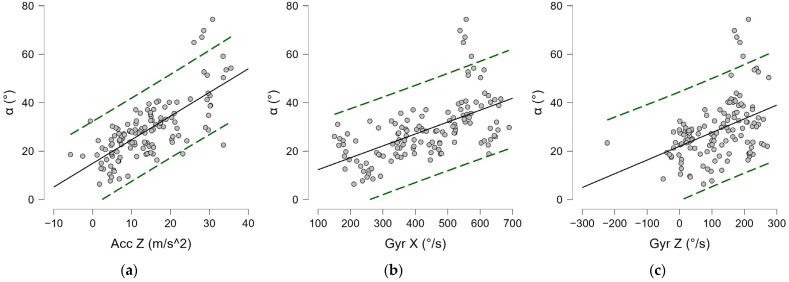
Spearman’s correlation (ρ) in the serve: (**a**) $\hat{\alpha }$—Acc Z; (**b**) $\hat{\alpha }$—Gyr X; (**c**) $\hat{\alpha }—$Gyr Z. Gyr X: trunk angular torsion velocity; Gyr Z: shoulder-over-shoulder angular velocity; Acc Z: horizontal acceleration; $\hat{\alpha }$: trunk angle. Green dashed lines indicate 95% predictions intervals.

**Figure 21:**
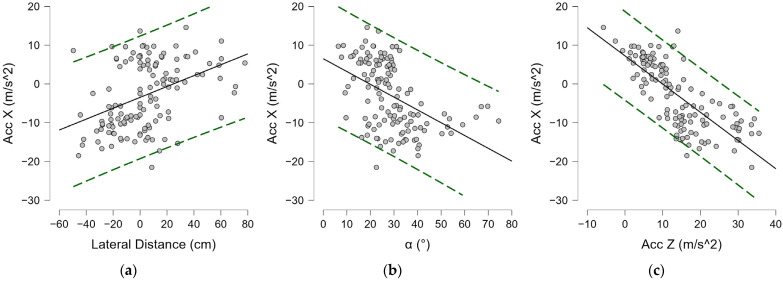
Spearman’s correlation (ρ) in the serve: (**a**) Acc X—Lateral distance; (**b**) Acc X—$\hat{\alpha }$; (**c**) Acc X—Acc Z. Lateral distance; Acc X: vertical acceleration; Acc Z: horizontal acceleration; $\hat{\alpha }$: trunk angle. Green dashed lines indicate 95% predictions intervals.

A medium–high correlation index was found between the lateral distance and trunk angle ($\hat{\alpha }$) of −0.556 (*p* < 0.001) (Figure 19a), and a more modest index was found with anteroposterior distance (ADP) (ρ = 0.211, *p* = 0.016) (Figure 19d). Moderate negative correlations were found between lateral distance and performance parameters (Acc Z, Gyr X, Gyr Z) (Figure 19), as reported in Table 7. Positive correlations, instead, were found between the latter and the angle ($\hat{\alpha })$, as shown in Figure 20 and reported in Table 7. Strong positive correlations were found between Gyr X (ρ = 0.871, *p* < 0.001) (Figure 19f) and Gyr Z acceleration (ρ = 0.708, *p* < 0.001) (Figure 19g). A positive mean correlation was found between Acc X values and lateral distance (Figure 21a), and a low but significant negative correlation was found with anteroposterior distance, as reported in Table 7. A strong negative correlation was found between the vertical acceleration (Acc X) and the horizontal acceleration (Acc Z) (ρ = −0.792, *p* < 0.001), as shown in Figure 21c, and the angular velocities Gyr X (ρ = −0.717, *p* < 0.001) and Gyr Z (ρ = −0.714, *p* < 0.001). In addition, a negative correlation was found between the trunk angle ($\hat{\alpha })$ and Acc X (Figure 21b), as reported in Table 7.

The best observed performances in terms of acceleration and angular velocity were also observed in the case of the serve at distances close to the average values. In particular, 3.37 cm for the LD, and 27.74 cm for the APD. These services corresponded to a frontal torso angle of about 34 degrees forward.

## 4. Discussion

In the present study, some biomechanical key points have been highlighted in two of the most important strokes in modern tennis: forehand and serve [12]. In particular, the investigated parameters have been the accelerations and torsion velocity of the trunk and shoulder extension in the serve (i.e., shoulder-over-shoulder), which are essential for maximizing racquet speed at ball impact [33,38]. In addition, some technical parameters, such as trunk angles and the—lateral and anteroposterior distance to the ball, have been analyzed.

Technique in tennis has several specific adaptations among each player [7]. For this reason, although structured in a controlled situation, the same gesture can differ from player to player [9]. In addition, as evidenced in the present study, a high variability occurs among repetitions of the same stroke by the same player [9], which is particularly pronounced in amateurs when compared with expert players [18]. Consequently, all of the parameters investigated presented high inter- and intra-group variability coefficients in both forehand and service strokes. For this reason, and due to the high number of variables that influence the technical gesture, the data did not show a normal distribution, despite the large number of shots examined (420 forehands and 130 services). Consequently, nonparametric tests were chosen for statistical inference and reliability. In both forehand and serve, the Spearman’s correlation coefficient between the first and second set of measurements was always strong and highly significant, or medium in the case of the trunk angle ($\hat{\alpha }$) in forehand only, while ICC was high in all of the parameters investigated. The coefficient of variation for repeated measurements was high (Table 2 and Table 6) for all of the parameters and extremely high (2684%) for the lateral distance in the serve due to the difference in the toss, showing how, for amateur players, the serve is strongly affected by the variability of the toss. However, Wilcoxon signed-rank *t*-test was not significant in the two sets for all parameters, resulting in the lack of any statistically significant differences between the two sets of measurements.

### 4.1. Forehand

In forehand, the lateral distance from the ball varies from player to player, particularly depending on the stance technique used. This variability required a separate analysis of the two techniques. Among all of the shots played, a significant correlation was found between the stance type, the lateral distance, and trunk torsion speed, which showed higher average and maximum levels in the open stance. A moderate positive correlation was found between the distance and trunk torsion speed in all forehand strokes (played open and neutral). The investigated parameters have been influenced, in a minor way, by the direction of the shot, years of experience, and the player’s height. In the controlled situation reproduced in the study, the neutral stance was preferred over the others (62.44%).

Although the neutral stance shows the least technical variance in the support [25,26], there was a considerable variation among each player in all of the assessed parameters. Nevertheless, the results show that a greater lateral distance can facilitate the transfer of horizontal energy to the ball through body weight shift and greater trunk twisting speed. The best observed performance in terms of horizontal acceleration, velocity, and angular velocity (higher than 80 percentile) occurred at ball distances between 79.26 and 120.31 cm. The optimal torso angles at which the best performance was expressed were between 1 and 30 degrees with respect to frontal tilt $\hat{\alpha }$ and in a range of ±10 degrees in the orientation of the shoulder line with respect to the net.

In open stances, a higher average value of lateral distance (128.71 cm) with respect to the neutral was found, with a smaller variability (14.6%). However, this is due to the measurement protocol used (because the nondominant foot was chosen as a reference). In this stance, the best performances are associated with lateral distances close to the mean or lower values (between 114 and 140 cm). The higher distances do not show high values of acceleration and angular velocity; consequently, they do not allow effective energy transfer to the ball.

A modest correlation, ranging from 0.281 to 0.432, was found between trunk tilt and orientation angle with respect to the lateral distance in forehand shots. It is worth noting that this finding was statistically significant despite all the other factors that affect ball distance, such as direction, playing experience, and player characteristics. For this reason, an analysis based on the collection of these data from the inertial sensor could also be used individually on each player (a situation that has already shown strong correlation indexes [65]) as an evaluation tool by which to identify the optimal distance. The best performance in the forehand stroke occurred with a torso tilt between 1° and 30° forward and a horizontal orientation ranging between approximately ±10° from the net at the impact.

### 4.2. Serve

Different lateral and anteroposterior distance distributions from the ball were found in players during the serve. The average placement of the ball toss agrees with what is reported in the literature for flat serves [20,38,39], i.e., forward aligned with the front foot or slightly to the right (for right-handed players). The mean values resulted in a lateral distance of 3.76 cm and an anteroposterior distance of 18.35 cm. At impact time, an average value of the torso flexion–extension angle $\hat{\alpha }$ of 28.60° was measured, with instantaneous values of the torsion speed of 430.85°/s and an angular shoulder-over-shoulder speed of 116.36°/s. A medium-to-high correlation index was found between the torso angle $\hat{\alpha }$ and the lateral distance of the ball at impact, suggesting that this varies with the ball toss.

From a performance point of view, excessive positive lateral ball distances (i.e., toward the right) worsen the expression of forward linear acceleration, angular torsion, and shoulder-over-shoulder velocity. However, a greater lateral distance was shown to increase the speed of upward thrust. A less advanced impact and ball toss also favored the upward thrust. As might logically have been expected, a strong negative correlation was found between upward and horizontal acceleration and trunk torsion velocity, indicating that greater upward thrust in one direction disfavors the other and vice versa.

The best observed performances in terms of acceleration and angular velocity were also observed in the case of the serve at distances close to the average values. These results suggest that, in amateur tennis service, a ball toss placed at a lateral distance of about 3 cm external from the front foot and an anteroposterior distance of about 28 cm inside the court can facilitate the sports task. The services in this range correspond to a frontal torso angle of about 34 degrees forward.

### 4.3. Applicability and Developments

The results from this study suggest that using a single IMU sensor applied via a chest strap makes it possible to obtain important information on key kinematic parameters in the forehand stroke and serve. In addition, because of the correlations found with the flexion–extension and orientation angles of the trunk in the hitting phase, the measurement of these could be used to give real-time feedback to players as an assessment tool. This system can find an important application, particularly in amateur tennis, where, as noted, there is a high variability in the technical execution of strokes, potentially acting as a guide to the execution of more biomechanically effective and efficient movements. In addition, technical improvements decrease the likelihood of the incurrence of injuries [8,18]. In all the game situations using the inertial sensor, a customized analysis for each player could be conducted to identify the most appropriate personalized suggestions for perfecting each player’s technique.

Depending on the type of player, the system could identify the optimal angles by which to favor greater angular torso twisting speed in slimmer and shorter players or to use greater acceleration and linear velocity in stronger individuals with long joint levers. The same procedure can be applied at the tactical level in the serve depending on whether the player wants to seek more margin at the net to reduce errors or to have more angle of incidence of the ball and make the first serve more effective by seeking more upward thrust. Conversely, the player could, for example, seek greater horizontal thrust in a grass tournament, resulting in a more effective serve, or look for increased trunk angular velocities to generate more ball spin. In this regard, further studies are necessary to investigate the application of this system to all service types. A similar procedure can be conducted in rebound shots as in the forehand, identifying the appropriate area of intervention according to each player’s tactical needs.

### 4.4. Limitations of the Study

Although the system was shown to be able to monitor the key parameters in the expression of ball speed, the outcome of the stroke, let alone its accuracy, was not analyzed and could be assessed in further studies. In addition, due to the need to standardize the investigation protocol, a single structured situation was examined for both shots, and not all possible game situations that may occur during a game were investigated. Furthermore, the impact of anatomical sensor placement could be investigated in future studies and opt for placement on the upper back [77]. Finally, although this system may find its main applicability in amateur tennis, the information derived from it does not provide a reference performance model.

## 5. Conclusions

The results of this study show high levels of reliability of the system adopted. By using a single inertial sensor mounted on a chest strap, which is minimally invasive and easy to use, it was possible to assess some of the most important kinematic parameters of movement that provide insight into the proper transfer of the kinetic chain. An appropriate lateral distance from the ball, especially one that is not too close in all stance types nor too far in open stances, allowed a greater expression of the kinematic parameters linked to a better performance in amateurs. In addition, the results show a medium correlation between distance to the ball and flexion–extension angle and orientation of the torso, despite the many factors that affect the ball distance, such as direction, playing experience, and player characteristics. As a result, this system can be proposed as a potential tool for obtaining information about the optimal ball distance or toss in the serve.

The potential use of a single inertial sensor to obtain this information and its ease of use opens the way for the development of real-time tennis technique evaluation systems. Further studies are also needed to investigate the application of this methodology to the other fundamentals of the game, such as the backhand and volley shots, and to analyze high-level players so as to provide important information related to the performance model as a reference for the development of technical skills.

## Figures and Tables

**Table 1:** Set-to-set repeatability of average lateral distance (LD) (cm), angular velocity (Gyr X) (°/s), horizontal acceleration (Acc Z) (m/s^2^), horizontal velocity (dv) (m/s), trunk angles ($\hat{\alpha }$) and azimuth ($\hat{Az}$) (°) of the forehands performed by 21 amateur tennis players. IQR, inter-quartile range; ρ, Spearman’s correlation coefficient; Wilcoxon signed-rank *t*-test; ES, effect size by the rank-biserial correlation; and 95% CI for ES. ** *p*-value < 0.01; *** *p*-value < 0.001.

	Set 1			Set 2			ρ	Wilcoxon-Signed Rank Test		ES	95% CI for ES
Parameters	Mean ± SD	Median	IQR	Mean ± SD	Median	IQR		W	*p*		
LD (cm)	95 ± 20	104	40	94 ± 20	104	38	0.894 ***	132	0.585	0.143	−0.331 to 0.559
Gyr X (°/s)	453 ± 154	450	287	470 ± 160	483	251	0.887 ***	105	0.733	−0.091	−0.522 to 0.377
Acc Z (m/s^2^)	13 ± 14	13	18	12 ± 15	13	17	0.969 ***	143	0.355	0.238	−0.241 to 0.624
dv (m/s)	0.10 ± 0.1	0.13	0.1	0.09 ± 0.1	0.14	0.1	0.973 ***	140	0.412	0.212	−0.266 to 0.607
$\hat{\alpha }$ (°)	9 ± 12	12	12	10 ±10	11	14	0.631 **	110	0.865	−0.048	−0.490 to 0.414
$\hat{Az}$ (°)	−104 ± 23	−118	29	−101 ± 21	−113	28	0.895 ***	71	0.128	−0.385	−0.717 to 0.085

**Table 2:** Set-to-set repeatability of average lateral distance (LD) (cm), angular velocity (Gyr X) (°/s), horizontal acceleration (Acc Z) (m/s^2^), horizontal velocity (dv) (m/s), trunk angles ($\hat{\alpha }$), and azimuth $\hat{Az}$ (°) of the forehands performed by 21 amateur tennis players. ICC_3,k_, intraclass correlation coefficient; 95% confidence interval (CI) for ICC; CV%, coefficient of variation for repeated measurements; 95% confidence interval (CI) for CV%; SEM, standard error of measurement; MDC, minimal detectable change based on a 95% confidence level.

Parameters	ICC_3,k_	95% CI for ICC	CV ^a^ %	95% CI for CV	SEM	MDC
LD (cm)	0.951	0.908 to 0.973	6.335	3.804 to 8.112	1.865	3.656
Gyr X (°/s)	0.955	0.917 to 0.976	9.500	0 to 13.517	13.349	26.165
Acc Z (m/s^2^)	0.987	0.976 to 0.993	17.067	9.054 to 22.374	0.694	1.359
dv (m/s)	0.985	0.973 to 0.992	16.453	8.732 to 21.568	0.007	0.014
$\hat{\alpha }$ (°)	0.834	0.690 to 0.911	82.146	26.412 to 113.130	1.788	3.505
$\hat{Az}$ (°)	0.967	0.939 to 0.982	6.145	3.392 to 8.001	1.669	3.271

^a^ Root mean square method.

**Table 3:** Spearman’s correlation (ρ) in the neutral stance forehand. Gyr X: trunk angular torsion velocity; Acc Z: horizontal acceleration; DV: horizontal velocity; $\hat{\alpha }$: trunk angle; $\hat{Az}$: azimuth.

	ρ	*p*
Lateral distance (cm)—Gyr X	0.568	<0.001
Lateral distance (cm)—Acc Z	0.487	<0.001
Acc Z—Gyr X	0.732	<0.001
$\mathrm{Lateral}\;\mathrm{distance}\;(\mathrm{cm})—\hat{Az}$	−0.402	<0.001
$\mathrm{Lateral}\;\mathrm{distance}\;(\mathrm{cm})—\hat{\alpha }$	0.432	<0.001
$\mathrm{Acc}\;Z—\hat{\alpha }$	0.472	<0.001
$\mathrm{Gyr}\;X—\hat{\alpha }$	0.527	<0.001
$\mathrm{dv}—\hat{Az}$	−0.599	<0.001
$\mathrm{Acc}\;Z—\hat{Az}$	−0.596	<0.001
$\mathrm{Gyr}\;X—\hat{Az}$	−0.410	<0.001

**Table 4:** Spearman’s correlation (ρ) in the open stance forehand. Gyr X: trunk angular torsion velocity; Acc Z: horizontal acceleration; dv: horizontal velocity; $\hat{\alpha }$: trunk angle; $\hat{Az}$: azimuth.

	ρ	*p*
Lateral distance (cm)—Acc Z	0.258	0.018
Lateral distance (cm)—dv	0.255	0.020
Gyr X—dv	0.879	<0.001
$\mathrm{Lateral}\;\mathrm{distance}\;(\mathrm{cm})—\hat{\alpha }$	−0.281	0.010
$\mathrm{Lateral}\;\mathrm{distance}\;(\mathrm{cm})—\hat{Az}$	−0.238	0.030
$\mathrm{Gyr}\;X—\hat{Az}$	−0.492	<0.001
$\mathrm{Acc}\;Z—\hat{Az}$	−0.560	<0.001
Acc Z—Gyr X	0.878	<0.001
$\mathrm{dv}—\hat{Az}$	−0.562	<0.001

**Table 5:** Set-to-set repeatability of average lateral distance (cm), angular velocity (Gyr X) (°/s), horizontal acceleration (Acc Z) (m/s^2^), horizontal velocity (dv) (m/s), trunk angles ($\hat{\alpha }$), and azimuth ($\hat{Az}$) (°) of the serves performed by 13 amateur tennis players. IQR, inter-quartile range; ρ Spearman’s correlation coefficient; Wilcoxon signed-rank *t*-test; ES, effect size by the rank-biserial correlation; and 95% CI for ES. *** *p*-value < 0.001.

	Set 1			Set 2			ρ	Wilcoxon-Signed Rank Test		ES	95% CI for ES
Parameters	Mean ± SD	Median	IQR	Mean ± SD	Median	IQR		W	*p*		
LD (cm)	6 ± 22	6	30	2 ± 22	1.3	34	0.879 ***	71	0.080	0.560	0.017 to 0.848
APD (cm)	20 ± 25	15	41	17 ± 24	13	37	0.901 ***	61	0.305	0.341	−0.256 to 0.749
Gyr X (°/s)	434 ± 149	400	236	428 ± 146	428	263	0.984 ***	51	0.735	0.121	−0.458 to 0.628
Gyr Z (°/s)	124 ± 85	132	162	110 ± 84	100	159	0.934 ***	69	0.110	0.516	−0.045 to 0.830
Acc Z (m/s^2^)	14 ± 8	12.5	11	14 ± 9	12.6	13	0.967 ***	47	0.946	0.033	−0.525 to 0.571
Acc X (m/s^2^)	−3 ± 8	−1.5	15	−3 ± 8	−1.2	17	0.973 ***	42	0.839	−0.077	−0.600 to 0.492
$\hat{\alpha }$ (°)	29 ± 14	27	14	28 ± 10	27	15	0.907 ***	35	0.497	−0.231	−0.692 to 0.364

**Table 6:** Set-to-set repeatability of average lateral distance (LD) (cm), angular velocity (Gyr X) (°/s), horizontal acceleration (Acc Z) (m/s^2^), horizontal velocity (dv) (m/s), trunk angles ($\hat{\alpha }$) and azimuth ($\hat{Az}$) (°) of the forehands performed by 21 amateur tennis players. ICC_3,k_*,* intraclass correlation coefficient; 95% confidence interval (CI) for ICC; CV%, coefficient of variation for repeated measurements; 95% confidence interval (CI) for CV%; SEM, standard error of measurement; MDC, minimal detectable change based on a 95% confidence level.

Parameters	ICC_3,k_	95% CI for ICC	CV ^a^ %	95% CI for CV	SEM	MDC
LD (cm)	0.973	0.941 to 0.988	2684%	0 to 4766	1.860	3.646
APD (cm	0.976	0.946 to 0.989	61%	0 to 89	2.049	4.015
Gyr X (°/s)	0.991	0.979 to 0.996	5%	3 to 7	7.719	15.130
Gyr Z (°/s)	0.963	0.918 to 0.984	260%	0 to 462	8.670	16.995
Acc Z (m/s^2^)	0.990	0.979 to 0.996	27%	0 to 46	6.108	11.971
Acc X (m/s^2^)	0.985	0.967 to 0.993	57%	0 to 93	0.527	1.033
$\hat{\alpha }$ (°)	0.955	0.899 to 0.980	10%	0 to 17	1.319	2.585

^a^ Root mean square method.

**Table 7:** Spearman’s correlation (ρ) in the serve. Gyr X: trunk angular torsion velocity; Gyr Z: shoulder-over-shoulder angular velocity; Acc Z: horizontal acceleration; ADP anterior–posterior distance; $\hat{\alpha }$: trunk angle.

	ρ	*p*
$\mathrm{Lateral}\;\mathrm{distance}\;(\mathrm{cm})—\hat{\alpha }$	−0.556	<0.001
Lateral distance (cm)—Gyr Z	−0.548	<0.001
Lateral distance (cm)—Gyr X	−0.373	<0.001
Lateral distance (cm)—ADP (cm)	0.211	0.016
Lateral distance (cm)—Acc Z	−0.460	<0.001
Lateral distance (cm)—Acc X	0.405	<0.001
Acc Z—Gyr X	0.871	<0.001
Acc Z—Gyr Z	0.708	<0.001
Acc Z—Acc X	−0.792	<0.001
ADP (cm)—Acc Z	0.262	<0.001
ADP (cm)—Acc X	−0.281	0.001
ADP (cm)—Gyr X	0.289	<0.001
Gyr X—Acc X	−0.717	<0.001
Gyr Z—Acc X	−0.714	<0.001
$\hat{\alpha }$—Acc Z	0.687	<0.001
$\hat{\alpha }$—Acc X	−0.517	<0.001
$\hat{\alpha }$—Gyr X	0.636	<0.001
$\hat{\alpha }$—Gyr Z	0.422	<0.001

## Data Availability

The raw data supporting the conclusions of this article will be made available by the authors on request.
